# Visceral Leishmaniasis Patients Display Altered Composition and Maturity of Neutrophils as well as Impaired Neutrophil Effector Functions

**DOI:** 10.3389/fimmu.2016.00517

**Published:** 2016-11-29

**Authors:** Endalew Yizengaw, Mulusew Getahun, Fitsumbrhan Tajebe, Edward Cruz Cervera, Emebet Adem, Getnet Mesfin, Asrat Hailu, Gert Van der Auwera, Vanessa Yardley, Mulualem Lemma, Ziv Skhedy, Ermias Diro, Arega Yeshanew, Roma Melkamu, Bewketu Mengesha, Manuel Modolell, Markus Munder, Ingrid Müller, Yegnasew Takele, Pascale Kropf

**Affiliations:** ^1^Department of Immunology, University of Gondar, Gondar, Ethiopia; ^2^Department of Medicine, Imperial College London, London, UK; ^3^Leishmaniasis Research and Treatment Centre, Gondar University, Gondar, Ethiopia; ^4^Department of Microbiology, Immunology and Parasitology, Addis Ababa University, Addis Ababa, Ethiopia; ^5^Department of Biomedical Sciences, Institute of Tropical Medicine, Antwerp, Belgium; ^6^Department of Immunology and Infection, London School of Hygiene and Tropical Medicine, London, UK; ^7^Department of Internal Medicine, University of Gondar, Gondar, Ethiopia; ^8^Department of Mathematics and Statistics, University of Hasselt, Hasselt, Belgium; ^9^Department of Cellular Immunology, Max-Planck-Institute for Immunobiology and Epigenetics, Freiburg, Germany; ^10^Third Department of Medicine (Hematology, Oncology, and Pneumology), University Medical Center Mainz, Mainz, Germany

**Keywords:** visceral leishmaniasis, neutrophils, neutrophil extracellular traps, reactive oxygen species, phagocytosis

## Abstract

Immunologically, active visceral leishmaniasis (VL) is characterized by profound immunosuppression, severe systemic inflammatory responses, and an impaired capacity to control parasite replication. Neutrophils are highly versatile cells, which play a crucial role in the induction as well as the resolution of inflammation, the control of pathogen replication, and the regulation of immune responses. Neutrophil functions have been investigated in human cutaneous leishmaniasis; however, their role in human VL is poorly understood. In the present study we evaluated the activation status and effector functions of neutrophils in patients with active VL and after successful anti-leishmanial treatment. Our results show that neutrophils are highly activated and have degranulated; high levels of arginase, myeloperoxidase, and elastase, all contained in neutrophils’ granules, were found in the plasma of VL patients. In addition, we show that a large proportion of these cells are immature. We also analyzed effector functions of neutrophils that are essential for pathogen clearance and show that neutrophils have an impaired capacity to release neutrophil extracellular traps, produce reactive oxygen species, and phagocytose bacterial particles, but not *Leishmania* parasites. Our results suggest that impaired effector functions, increased activation, and immaturity of neutrophils play a key role in the pathogenesis of VL.

## Introduction

Leishmaniases are caused by a group of vector-borne parasites that represent a major public health problem worldwide, affecting 98 countries (1). The diseases present with a wide range of symptoms, ranging from cutaneous leishmaniasis (CL) to the most severe form of the disease, visceral leishmaniasis (VL). An estimated 0.2–0.4 million new cases of VL and 50,000 deaths occur each year; however, due to vast under-reporting of cases, these numbers are likely to be a significant underestimation of the real burden (1, 2). The majority of VL cases occur in Bangladesh, India, Nepal, South Sudan, Sudan, Ethiopia, and Brazil (2). VL is caused by *Leishmania donovani* or *Leishmania infantum* parasites that are transmitted during the blood meal of infected sand flies, and migrate from the skin to internal organs such as spleen, liver, and bone marrow. The majority of individuals infected with these parasites will be able to control infection; however, some will develop symptomatic disease, in which the mortality rate can be as high as 100% in untreated patients. The symptoms include fever, weight loss, severe anemia, hepato- and splenomegaly, and pancytopenia. However, the factors accounting for the development of symptomatic disease or control of VL are poorly understood. It is generally accepted that active VL is associated with strong immune suppression, as shown by the impaired capacity of peripheral blood mononuclear cells (PBMCs) to proliferate and produce IFN-γ [reviewed in Ref. (3, 4)]. Recently, this notion was challenged by studies performed in India (5, 6): using a whole blood assay, the authors showed that CD4^+^ T cells produce similar levels of IFN-γ that can limit *Leishmania* parasite replication during active VL. In contrast, our recent work performed in Ethiopia showed that whole blood cells produce no or low IFN-γ, suggesting that whole blood cells from VL patients in Ethiopia are hyporesponsive (7).

One key feature of patients with active VL is the high plasma level of pro-inflammatory cytokines and chemokines such as TNF-α, IFN-γ, IL-1, IL-6, IL-8, IL-12, and IP-10 [reviewed in Ref. (3, 4)]; all characteristic of a systemic and acute inflammatory response, similar to that observed in diseases such as severe malaria and sepsis (8, 9). Neutrophils are one of the main mediators of inflammation, they are the first cells to be recruited to the site of inflammation and can eliminate pathogens via several mechanisms; including phagocytosis, production of toxic molecules such as reactive oxygen species (ROS), anti-bacterial proteins, and neutrophil extracellular traps (NETs), that function by killing and/or containing pathogens (10, 11). They also play a crucial role in the resolution of inflammation by scavenging cytokines, producing pro-resolving mediators and undergoing apoptosis (10, 11). However, the serine proteases contained in the granules of neutrophils can also cause excessive tissue damage [reviewed in Ref. (12)]. Neutrophil function in human CL has been investigated in several studies (13–15) and they indicate that neutrophils are active players in patients with both acute and chronic CL and are important immune regulators that can have beneficial as well as detrimental effects (16). Since different *Leishmania* species can drive distinct neutrophil functions (17), it is of utmost importance that the phenotypes and functions of neutrophils isolated from patients infected by different *Leishmania* species are investigated. In VL patients, several studies have pointed to a possible role of inflammation in the pathogenesis of VL (18, 19); however, the role of neutrophils in human VL is poorly understood. Previous studies have shown that neutrophils from healthy donors have the ability to phagocytose and kill *L. donovani* (20). The killing mechanisms of intracellular *Leishmania* parasites in human neutrophils have not been fully identified; *L. donovani* phosphatase can suppress superoxide anion production, suggesting that this might impair the ability of neutrophils to kill parasites (21). In addition, *Leishmania*-containing phagosomes in neutrophils avoid fusion with granules involved in acidification of vacuoles, thereby contributing to parasite survival (22). *L. donovani* (23) and *L. infantum* (24) both can induce the release of NETs; however the survival of *L. donovani* was not compromised (23), whereas *L. infantum* was being killed at least in part via the activity of 3'-nucleotidase/nuclease (24). VL patients are severely neutropenic, and indeed, bacterial infections are common in these patients and are the primary cause of deaths (25). The severe neutropenia associated with VL has been shown to be reversed by GM-CSF, which resulted in reduced secondary infections, further emphasizing the role of neutrophils during VL (26).

In the present study, we characterized the activation status and effector functions of neutrophils during active VL and following successful treatment.

## Materials and Methods

### Subjects and Sample Collection

The study was approved by the Institutional Review Board of the University of Gondar (IRB, reference SBMLS/Dec. 199/07). For this cross-sectional study, non-endemic male controls with no prior history of VL were recruited amongst the hospital staff; male VL patients were recruited from the Leishmaniasis Treatment and Research Center of the Gondar University Hospital before and after treatment. Informed written consent was obtained from each patient and control. The exclusion criteria were age (<18 years) and coinfection with tuberculosis, malaria, and HIV. The diagnosis of VL was based on positive serology (rK39) and the presence of amastigotes in spleen or bone marrow aspirates (27). Patients were treated with a combination of sodium stibogluconate (SSG, 20 mg/kg body weight/day) and paromomycin (PM, 15 mg/kg body weight/day) injections, given intramuscularly for 17 days or with Ambisome^®^ (max of 30 mg/kg body weight, with six injections of 5 mg/kg body weight/day). There was an initial clinical cure (ICC) rate of 100% after treatment; ICC was defined as follows: at the end of treatment, patients look improved, afebrile, and have a smaller spleen size than on admission and have an increased hemoglobin (Hgb) level.

Six milliliter of blood was collected in heparin tubes and was processed within 10 min after collection: following density gradient centrifugation on HistopaqueH-1077 (Sigma), the plasma was isolated from the top layer and neutrophils from the erythrocyte fraction by dextran sulfate sedimentation (28), and were used immediately. The blood from all three groups (healthy controls, VL patients and treated patients) was treated in the same way and within a maximum period of time of 2.5 h from the time the individuals were bled to the time samples were run on the flow cytometer.

It was sometimes difficult to obtain samples from all patients, for two main reasons: poor medical conditions of the patients at arrival in the hospital and/or frequent electricity cuts that did not allow for immediate processing of the blood. In addition, due to the severe pancytopenia of VL patients, and the small amount of cells recovered, it was not always possible to do all tests.

### *Leishmania donovani* Parasites

GFP-labeled *L. donovani* (Dr. V. Yardley, LSHTM) was used for the phagocytosis assays and *L. donovani* isolated from a spleen biopsy obtained from a patient with active VL was used to determine parasite-induced production of ROS. The species of the GFP-labeled *Leishmania* strain was confirmed to be *L. donovani* by means of heat-shock protein 70 gene sequence analysis (29).

### Flow Cytometry

#### Characterization of Neutrophil Activation

The following antibodies were used to assess the activation status of neutrophils by flow cytometry: anti-CD10 (clone eBioSN5c), anti-CD11b (clone ICRF44), anti-CD13 (clone WM-15), anti-CD15 (clone HI98), anti-CD16 (clone eBioCB16), anti-CD33 (clone WM53), anti-CD62L (clone DREG-56), anti-CD66b (clone G10F5) (eBioscience), and anti-arginase I [HyCult Biotechnology: clone 6G3, labeled as described in Ref. (30)]. Cells were incubated with antibody concentration determined in preliminary experiments, washed with PBS, the fixation step was performed with 2% formaldehyde in PBS and the permeabilization step with 0.5% saponin in PBS. Median Fluorescence Intensity (MFI) was used to demonstrate increase or decrease in expression of the markers. The percentages for the isotype controls were <1%. At least 2,500 CD15^+^ cells were collected for each sample.

### Phagocytosis

Uptake of pHrodo^®^ Green *Escherichia coli* BioParticles^®^ Conjugate (Molecular probes™) was used to assess the phagocytic capacity of neutrophils, as described in the manufacturer’s protocol.

Uptake of parasites was assessed using GFP-labeled *L. donovani*: the parasites were first incubated for 15 min in decomplemented human plasma; next, 1 × 10^5^ neutrophils were cocultured with 5 × 10^6^ GFP-labeled *L. donovani* at 37°C with 5% CO_2_. After 60 min, neutrophils were washed twice in PBS. Trypan blue was added just before flow cytometry analyses to quench the fluorescence of non-internalized parasites (31).

### ROS Production

Total ROS detection kit (Enzo) was used to evaluate the production of ROS by neutrophils, as described in the manufacturer’s protocol.

Flow cytometry acquisition was performed using a FACSCalibur (BD Biosciences) and data were analyzed using Summit v4.3 software.

### Total, Band, and Segmented Neutrophil Counts

Total neutrophil counts were obtained using a CELL-DYN 1800 (Abbott). Smears of purified neutrophils were stained with Giemsa and the percentages of band and segmented neutrophils per 100 neutrophils were counted microscopically.

### ELISA

IL-17A and IL-27 were measured in plasma with Ready-SET-Go! ELISA kits (eBioscience). Myeloperoxidase and elastase levels in plasma were measured using ELISA kits (BioVendor R&D). The ability of neutrophils to release NETs was assessed by measuring the NET-associated elastase using NETosis assay kit (Cayman Chemical). All ELISA were performed according to the manufacturers’ protocol.

### Arginase Activity

The enzymatic activity of arginase was measured as previously described (32). To determine arginase activity in the plasma, urea concentrations were first determined in the plasma, without the activation and hydrolysis steps; these values were subtracted from those obtained by measuring the urea.

One unit of enzyme activity is defined as the amount of enzyme that catalyzes the formation of 1 mmol of urea per minute.

### Statistical Analysis

Data were evaluated for statistical differences using a Wilcoxon matched-pairs tests or Kruskal–Wallis test (GraphPad Prism 6) and differences were considered statistically significant at *p* < 0.05. Unless otherwise specified, results are expressed as median.

## Results

### Characterization of Neutrophils in Patients with Active VL

**Figure 1:**
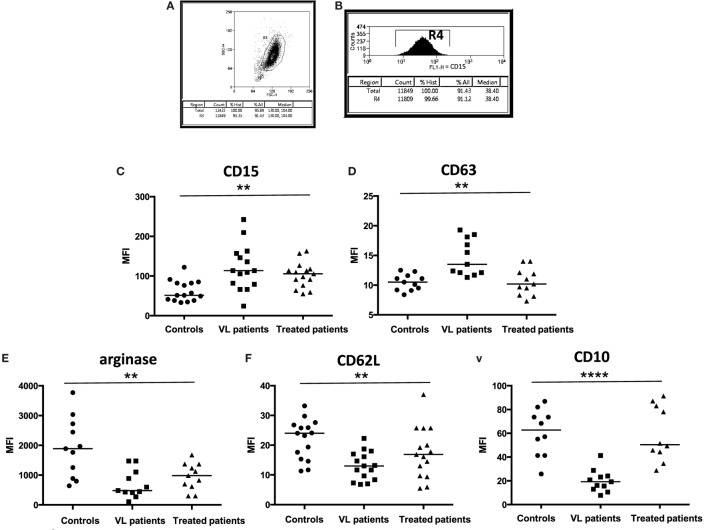
**Activation status of neutrophils**. **(A)** Neutrophils were isolated by dextran sulfate sedimentation from the blood of controls, VL, and treated patients (=VL patients after successful treatment, with initial clinical cure) and **(B)** the frequencies of CD15^+^ cells were determined by flow cytometry. The expression levels (MFI = Median Fluorescence Intensity) of CD15 [**(C)**, 15 individuals in each group], CD63 [**(D)**, 11 individuals in each group], arginase [**(E)**, 11 individuals in each group], CD62L [**(F)**, 15 individuals in each group], and CD10 [**(G)**, 10 controls, 11 VL, and 10 treated patients] were determined in the cells from gate R4 by flow cytometry. Statistical differences were determined using a Kruskal–Wallis test.

Neutrophils are major players in the induction, maintenance, and resolution of inflammation (11). However, the role of neutrophils in active VL, a disease characterized by high systemic inflammation, is poorly understood. Neutrophils were purified by dextran sulfate sedimentation (>95% of the cells in the live gate, Figure 1A) and the purity of neutrophils (defined as CD15^+^) was >99%, Figure 1B. We analyzed the activation status of neutrophils by measuring the levels of intracellular and surface markers of neutrophils that had been previously associated with activation (33). As shown in Figure 1, the MFIs of CD15 were significantly increased in VL patients (*p* = 0.0022) and in treated patients (*p* = 0.0122), as compared to controls. CD63 was also increased in VL patients (*p* = 0.0050) and decreased significantly after treatment (*p* = 0.0063). The MFI of intracellular arginase and the MFI of CD62L and CD10 were all decreased in neutrophils from VL patients (Figure 1, *p* = 0.0028, *p* = 0.0037, and *p* = 0.0007, respectively); whereas the MFIs of arginase and CD62L did not increase significantly (*p* > 0.05) following successful treatment of VL patients, CD10 expression was significantly increased (*p* = 0.0006). No significant differences were found in the MFIs of CD13, CD16, CD33, and CD66b (data not illustrated).

The results presented in Figure 1 show that neutrophils are significantly more activated in VL patients.

### Maturation Stage of Neutrophils

**Figure 2:**
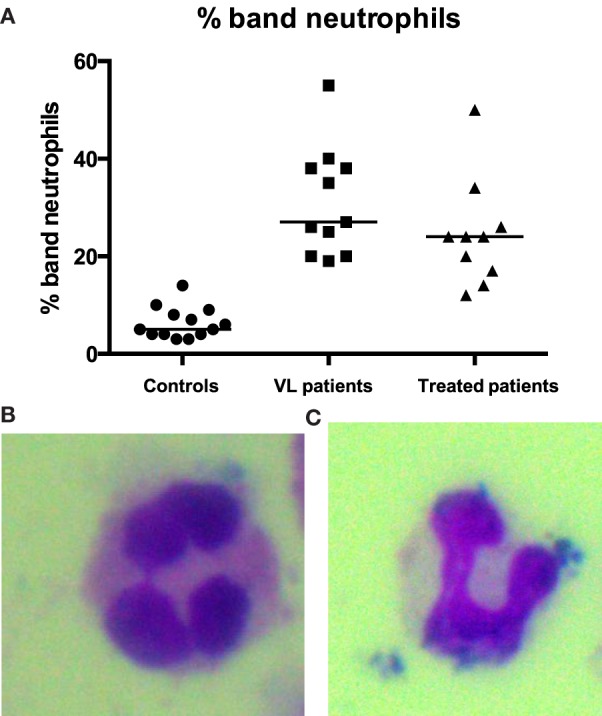
**Percentages of band neutrophils**. Smears from PBMCs and neutrophils purified from the blood of 13 controls, 11 VL, and 10 treated patients (=VL patients after successful treatment, with initial clinical cure) by double density gradient centrifugation were stained with Giemsa and the percentages of segmented **(B)** and band **(A,C)** neutrophils were counted per 50 neutrophils. Statistical differences were determined using a Kruskal–Wallis test.

Since CD10 is present on immature band neutrophils (34), we estimated the maturation stage of neutrophils. Smears of purified neutrophils from controls, VL, and treated patients were stained with Giemsa and the numbers of segmented (Figure 2B) and band (Figure 2C) neutrophils were counted. Results presented in Figure 2A show that the frequency of band neutrophils is significantly higher in VL patients than in controls (*p* < 0.0001), and this did not change significantly after treatment. These results show that a large proportion of the neutrophils in the blood of VL patients are immature cells.

### Inflammatory Cytokine Profile and Evaluation of Secreted Granule Content of Neutrophils

**Figure 3:**
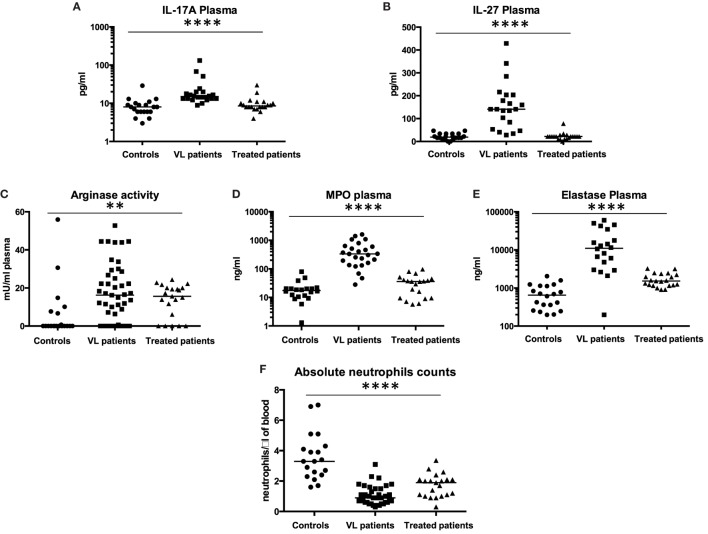
**IL-17A, IL-27, arginase, myeloperoxidase, and elastase levels in plasma**. Plasma was isolated from the blood of healthy controls, VL, and treated patients (=VL patients after successful treatment, with initial clinical cure) and the levels of **(A)** IL-17A and **(B)** IL-27 were measured by ELISA (20 controls, 22 VL, and 20 treated patients). The activity of arginase [**(C)**, 20 controls, 45 VL, and 21 treated patients], the levels of MPO [**(D)**, 25 controls, 20 VL, and 20 treated patients], and elastase [**(E)**, 20 controls, 20 VL, and 21 treated patients] were measured by ELISA as described in Section “Materials and Methods.” The neutrophil counts **(F)** were measured using a CELL-DYN 1800, Abbott. Statistical differences were determined using a Kruskal–Wallis test.

IL-17A is associated with neutrophilic inflammation and indeed, our results show that IL-17A is significantly increased in the plasma of VL patients (Figure 3A, *p* < 0.0001). Since a recent study suggested that, in an experimental model of VL, IL-27 mediates susceptibility to VL by suppressing IL-17, we measured the levels of IL-27 in the plasma of our three cohorts of individuals. As shown in Figure 3B, the levels of IL-27 were significantly increased in VL patients (*p* < 0.0001).

Arginase, myeloperoxidase, and elastase are present in primary granules of neutrophils, and are the last granules to be released upon activation of neutrophils. To further characterize the activation status of neutrophils, the levels of arginase, myeloperoxidase, and elastase were measured in the plasma of controls, VL, and treated patients. Arginase activities, myeloperoxidase, and elastase levels were all significantly increased in plasma from VL patients (Figure 3C, *p* = 0.0024; Figure 3D, *p* < 0.0001; and Figure 3E, *p* < 0.0001, respectively); the levels of arginase, myeloperoxidase, and elastase decreased significantly after anti-leishmanial treatment (Figure 3A, *p* = 0.0392, *p* < 0.0001, and *p* = 0.0093, respectively).

To determine whether these elevated levels of arginase, MPO, and elastase might be due to increased neutrophil counts in VL patients, the absolute neutrophil counts were measured and as shown in Figure 3F, it was strikingly lower in VL patients (*p* < 0.0001) and increased significantly (*p* = 0.0159) after successful treatment, while still staying significantly lower than those from controls (*p* = 0.0034).

These results show that levels of arginase, MPO, and elastase are elevated in the plasma of VL patients and further confirm that VL patients are highly neutropenic.

### Effector Functions of Neutrophils

Neutrophils play a major role in controlling pathogens by releasing NETs, acting as phagocytic cells, and producing ROS (11). Next we assessed these effector functions in neutrophils isolated from controls, VL, and treated patients. Neutrophils were purified by dextran sulfate sedimentation (>95% of the cells in the live gate, Figure 1A). We analyzed the following neutrophil (as defined by CD15^+^ cells, >99%, Figure 1B) effector functions.

### NETosis

**Figure 4:**
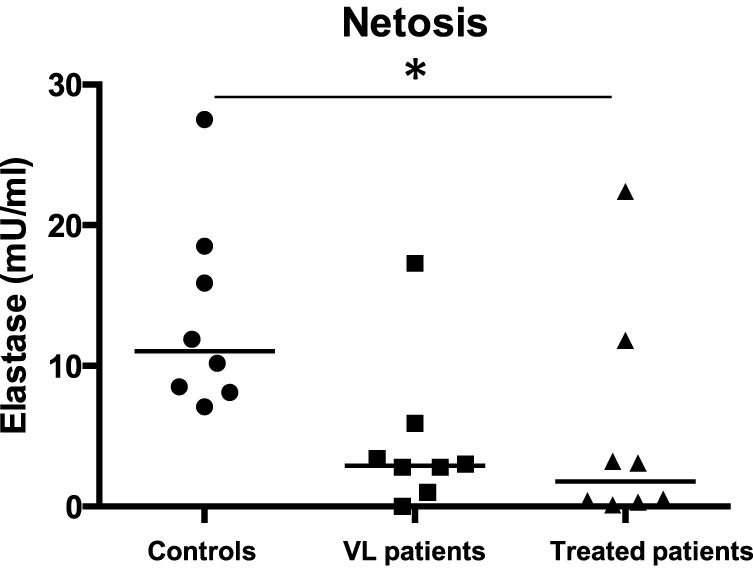
**NETosis**. Neutrophils were isolated by dextran sulfate sedimentation from the blood of eight controls, eight VL, and eight treated patients (=VL patients after successful treatment, with initial clinical cure). Neutrophils were activated with PMA to induce NET release and the NET-associated elastase was measured by ELISA as described in Section “Materials and Methods.” Statistical differences were determined using a Kruskal–Wallis test.

Purified neutrophils were induced to release NETs by PMA and as shown in Figure 4, neutrophils from VL patients have an impaired capacity to undergo NETosis as compared to controls (*p* = 0.0399); this was maintained even after successful treatment.

### Phagocytosis

**Figure 5:**
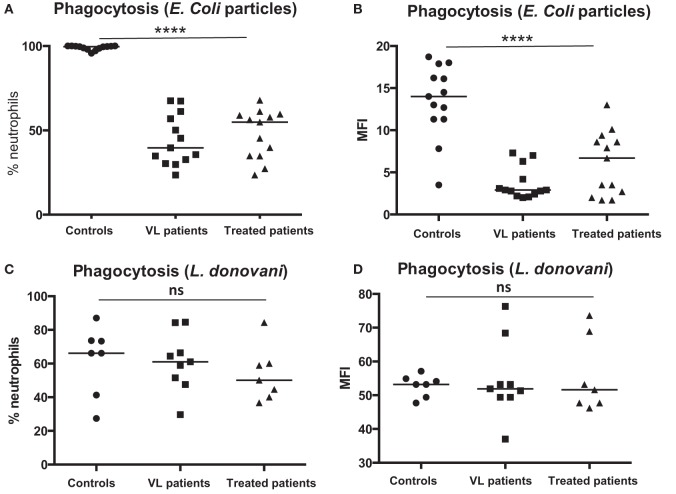
**Phagocytosis of particles and *L. donovani* parasites**. Neutrophils were isolated by dextran sulfate sedimentation from the blood of controls (13 individuals for *E. coli* and seven individuals for *L. donovani*), VL patients (13 individuals for *E. coli* and nine individuals for *L. donovani*), and treated patients (=VL patients after successful treatment, with initial clinical cure) (=VL patients after successful treatment, 13 individuals for *E. coli* and seven individuals for *L. donovani*). The percentages of neutrophils (defined as CD15^+^ cells, Figure 1B) phagocytosing *E. coli* particles **(A)**, the intensity (MFI = Median Fluorescence Intensity) of *E. coli* particles in neutrophils **(B)**, the percentages of neutrophils (defined as CD15^+^ cells, Figure 1B) phagocytosing GFP-labeled *L. donovani* **(C)**, and the intensity of GFP-labeled *L. donovani* parasites in neutrophils **(D)** were measured by flow cytometry. Statistical differences were determined using a Kruskal–Wallis test.

Next, we assessed the capacity of neutrophils isolated from controls, VL, and treated patients to phagocytose particles and GFP-labeled *L. donovani*. Our results show that a significantly lower percentage of neutrophils (Figure 5A) isolated from VL patients phagocytosed significantly less particles (Figure 5B, *p* < 0.0001) as compared to controls; this was not significantly higher following successful treatment.

Next, the capacity of neutrophils to phagocytose GFP-labeled *L. donovani* parasites was assessed. As shown in Figures 5C,D, a similar percentages of neutrophils (Figure 5C) phagocytosed a similar number of parasites (Figure 5D).

These results show that neutrophils isolated from VL patients have an impaired capacity to phagocytose *E. coli* particles, but not *L. donovani* parasites.

### ROS Production

**Figure 6:**
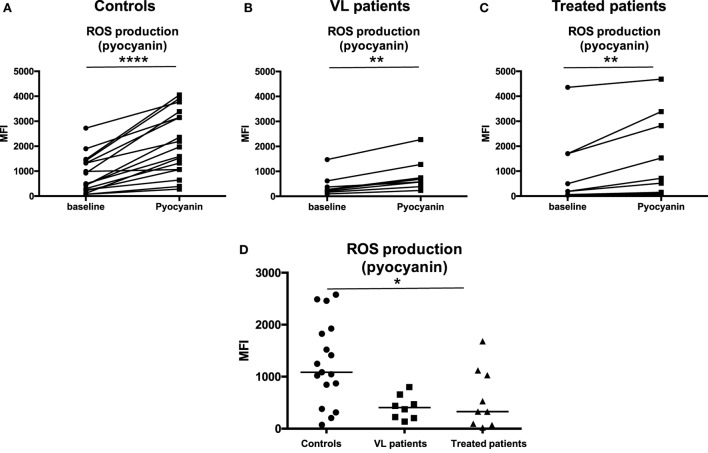
**ROS production in response to pyocyanin**. Neutrophils were isolated by dextran sulfate sedimentation from the blood of 17 controls **(A)**, eight VL **(B)**, and nine treated patients [=VL patients after successful treatment, with initial clinical cure, **(C)**] and were incubated in the absence (baseline) or in the presence of pyocyanin. The production of ROS was evaluated by measuring the fluorescence (MFI = Median Fluorescence Intensity) resulting from the reaction of ROS with the Oxidative Stress Detection buffer. **(D)** MFI values were obtained by subtracting the values of obtained from the neutrophils (defined as CD15^+^ cells, Figure 1B) incubated without pyocyanin (baseline) from those incubated in the presence of pyocyanin. Statistical differences were determined using a Wilcoxon **(A–C)** or a Kruskal–Wallis test **(D)**.

Results in Figure 6 show that neutrophils from controls, VL, and treated patients produced increased levels of ROS following activation with pyocyanin (Figures 6A–C, *p* > 0.0001, *p* = 0.0078, *p* = 0.0039, respectively); however, as shown in Figure 6D, increase in ROS production in response to pyocyanin was significantly lower in VL patients (*p* = 0.0424) as compared to controls and this did not increase in treated patients (*p* > 0.05).

**Figure 7:**
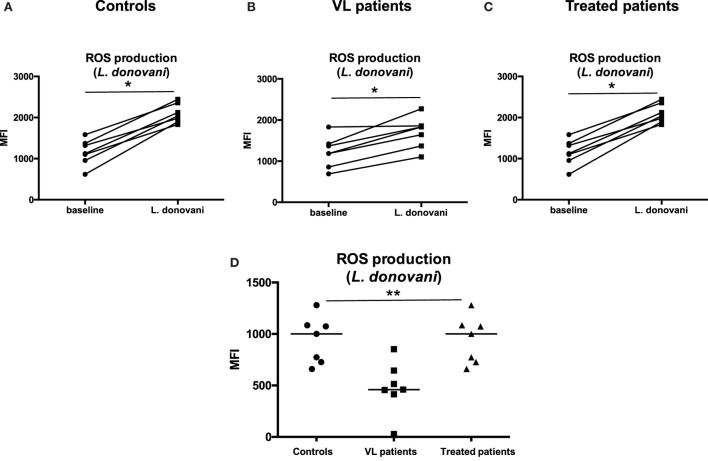
**ROS production in response to *L. donovani* parasites**. Neutrophils were isolated by dextran sulfate sedimentation from the blood of seven controls **(A)**, seven VL **(B)**, and seven treated patients [=VL patients after successful treatment, with initial clinical cure, **(C)**] and were incubated in the absence (baseline) or in the presence of *L. donovani* parasites. The production of ROS was evaluated by measuring the fluorescence (MFI = Median Fluorescence Intensity) resulting from the reaction of ROS with the Oxidative Stress Detection buffer. **(D)** MFI values were obtained by subtracting the values of obtained from the neutrophils (defined as CD15^+^ cells, Figure 1B) incubated without *L. donovani* (baseline) from those incubated in the presence of *L. donovani* parasites. Statistical differences were determined using a Wilcoxon **(A–C)** or a Kruskal–Wallis test **(D)**.

Next we measured the production of ROS following infection of neutrophils with *L. donovani* parasites. As shown in Figures 7A–C, *L. donovani* infection induced an increase in ROS production in the neutrophils isolated from all three groups (*p* = 0.0156); however, this increase was significantly lower in VL patients (Figure 7D, *p* = 0.0161); this improved after successful treatment (Figure 7D, *p* = 0.0161).

These results show that neutrophils isolated from VL patients have an impaired capacity to produce ROS.

## Discussion

In the present study we aimed to determine the frequency, activation status, maturation stage, and function of neutrophils in patients with active VL in Gondar, Northwest Ethiopia. We provide evidence that these cells are activated, as shown by the increased levels of CD15 and CD63 and decreased levels of intracellular arginase, CD62L, and CD10. Whereas CD15 and CD62L are not directly related to neutrophil effector functions, modulation of their expression levels can be used as a marker of activation: CD62L is known to be shed and lost from the cell surface of neutrophils following activation (34). CD15 is present in the membranes of the primary and secondary granules, and is upregulated on neutrophils following activation (34). We have previously shown that CD15 and CD63 are markers of activation as they are upregulated on a subpopulation of activated neutrophils in the blood of healthy controls, as well as the blood from HIV seropositive patients (33), VL patients (35, 36), cord blood, pregnant women, and in placentae (37). CD63 is found in the primary granules of neutrophils and becomes upregulated at the surface of neutrophils during degranulation (28, 36, 38). Indeed, our results show that arginase, an enzyme also found in the primary granules of neutrophils, is significantly lower in neutrophils; suggesting that these cells have been activated and have degranulated. The increased levels of arginase, myeloperoxidase, and elastase we measured in the plasma of VL patients further support this conclusion; these enzymes are all contained in azurophilic granules that are released following activation of neutrophils (38, 39). These enzymes could also exacerbate immunopathological processes and therefore disease severity by the following mechanisms:

(i) Arginase: we have previously shown that arginase-induced l-arginine depletion results in T cell suppression (35, 36, 40, 41) and this mechanism is therefore likely to contribute to T cell hyporesponsiveness in VL patients. Furthermore, the catabolism of l-arginine by arginase results in the production of polyamines that promotes parasite growth (32), and might therefore contribute to the unrestricted parasite replication in patients with active VL.
(ii) Myeloperoxidase is a major player in the oxidative killing by neutrophils (42) and is therefore likely to contribute to parasite killing; however, little is known about the actual effects of MPO on the fate of intracellular *Leishmania* parasites. One study showed that myeloperoxidase affects parasite survival (43); however, a recent study showed that following phagocytosis of *Leishmania* parasites by neutrophils, the fusion of the myeloperoxidase containing granules with phagosomes is blocked, thereby preventing potential toxic effects of the content of these granules (22). Besides, the increased level of MPO in the plasma is not an indication of parasite killing by neutrophils, since this needs to take place intracellularly; however, the increased MPO level in the plasma is a clear indicator of neutrophil activation.
(iii) Elastase was also significantly increased in the plasma of VL patients. It has been recently shown that neutrophil elastase contributes to parasite killing via crosstalk with toll-like receptors (TLRs) on macrophages (44). Since *Leishmania* parasites are not efficiently killed, elastase may have been produced in excess as a mechanism of feedback control to improve parasite killing. However, we have not tested the impact of elastase or MPO released by neutrophils on parasite killing. Furthermore, neutrophil-derived elastase is a potent mediator of inflammation and the increased levels we detected in the plasma are likely to contribute to the high and sustained systemic inflammation in patients with active VL and might be a potential therapeutic target (45). Of note, we cannot exclude that elastase and to a lesser extent myeloperoxidase we detected in the plasma has also been produced by macrophages. In addition to their potential role in T cell responses and parasite killing, the elevated levels of arginase, MPO, and elastase could be considered as biomarkers of a severe and systemic inflammatory response that is at least in part caused by dysregulated neutrophil homeostasis and high neutrophil activation.

Our results further confirm that the levels of IL-17A and IL-27 are elevated in the plasma of patients with active VL (19, 46–48). This is line with the increased mixed cytokine and chemokine profile characteristic of VL patients (3). In experimental models of leishmaniasis, the roles of IL-17A and IL-27 are still controversial: IL-17A, in synergy with IFN-γ, has been shown to potentiate leishmanicidal activities of infected macrophages (49). In contrast, IL-17 can promote progression of CL in susceptible mice (50). The parasite load in IL-27R^−−^ mice infected with *Leishmania major* was not affected (51), whereas IL-27 has been shown to promote *Leishmania amazonensis* replication in macrophages (52). Interestingly, a recent study showed that IL-27 could mediate susceptibility of mice to *L. infantum* infections by suppressing IL-17 production by neutrophils (53).

A recent study (54) suggested that a subpopulation of low-density neutrophils (36) express markers of antigen-presenting cells. Whereas the authors could not demonstrate that these neutrophils can stimulate T cells, it cannot be excluded that these cells could act as antigen-presenting cells.

Neutropenia is one of the main clinical characteristics of patients with active VL and indeed, here we show drastically reduced numbers of neutrophils in VL patients. This supports a previous report indicating that neutrophil reserve in the bone marrow of VL patients is markedly reduced (55). In addition, the expression levels of CD10 are significantly reduced on the surface of neutrophils. CD10, also known as Common Acute Lymphocytic Leukemia antigen (CALLA), is a neutral endopeptidase and is a member of the metalloprotease family; it is expressed on B and T cell precursors, and neutrophils. CD10 is important in the regulation of chemotactic and inflammatory processes involving neutrophils. It is also used as a marker of neutrophil maturity as it is only expressed on immature band neutrophils (34). Furthermore, newly released neutrophils express much less CD10 (56). Our results show a dramatic reduction in the expression levels of CD10 on neutrophils. It is also possible that the decreased levels of CD10 might be a sign of activation, and indeed, this has been previously described for low-density granulocytes from SLE patients (57) as well as following *in vivo* inflammatory challenges (58). Since CD10 is a major metalloproteinase that can regulate levels of biologically active peptides that initiate inflammation, it is possible that CD10 is downregulated on the surface of neutrophils in VL patients in an attempt to control the severe inflammatory response.

The immaturity of neutrophils in VL patients might also explain why these cells, despite the fact that they can be activated, display impaired effector functions. Indeed, our results show that neutrophils from VL patients cannot produce ROS efficiently, both following activation with pyocyanin and *L. donovani*. This might not only contribute to the lack of parasite killing, but also explain the increased incidence of bacterial infections in these patients (25). Furthermore, our results show that neutrophils display an impaired capacity to phagocytose *E. coli* particles, further explaining the high incidence of secondary infection in VL patients. In contrast, neutrophils from VL patients had a similar ability to phagocytose *L. donovani* parasites as compared to healthy controls. This could facilitate the survival and propagation of *L. donovani*: phagocytosis of infected apoptotic neutrophils by macrophages can result in a “silent” entry, without activation and therefore avoid killing of the parasites by the macrophages, thereby promoting uncontrolled parasite replication. It has also been shown that *Leishmania*-infected macrophages have a prolonged half-life, this could allow more time for the monocytes to migrate to the site of infection and uptake of infected apoptotic neutrophils (59, 60).

Phagocytosis of *Leishmania* parasites by neutrophils has been shown to be dependent on complement receptor 3 (CR3) and TLR2, suggesting both opsonin-dependent and opsonin-independent mechanisms are involved (61, 62). Lipophosphoglycan (LPG) also plays important roles in the early interaction of *Leishmania* parasites with phagocytic cells: LPG has been shown to inhibit activation of protein kinase C (PKC), a key macrophage signaling protein, and help in the parasite’s silent entry (63).

Importantly, our results also show that the majority of parameters we analyzed such as the activation status of neutrophils, the levels of arginase in plasma, the total neutrophil counts, the number of band neutrophils, NETosis, ROS production, and phagocytosis of *E. coli* particles were not restored after successful treatment of VL. These results show that despite the “clinical cure,” the immune system of these patients is still impaired at the time they are released from hospital. This is in line with our previous study that showed that the capacity of whole blood cells to respond to antigenic and polyclonal stimulation with IFN-γ production was not restored in treated patients (7). This is of relevance in Ethiopia, where a new treatment is used, a combination of PM and SSG, which reduced the hospitalization of VL patients from 28 to 17 days. However, this combination treatment has been associated with increased rates of relapse of VL in Sudan (64). This will also be of particular relevance for VL patients coinfected with HIV that have a significantly higher risk of relapse (65) following treatment.

Whereas the role of neutrophils in human VL has been poorly characterized, the role of neutrophils in CL has been better defined, primarily in experimental model of CL. For example, neutropenia induced transiently by depletion of neutrophils with monoclonal antibodies or in genetically modified mice without mature neutrophils (Genista mice) results in uncontrolled parasites replication following infections with *Leishmania mexicana* (66). NETosis has been shown to be induced *in vitro* by *L. amazonensis, L. major*, and *L. mexicana* (17, 66, 67), with killing of *L. amazonensis* (67), but not *L. mexicana* (66). ROS production has also been shown to differ depending on the strain of parasites: production of ROS was significantly higher following infection of neutrophils with *Leishmania braziliensis* as compared to *L. amazonensis* (68). Altogether, the results obtained in both human and experimental leishmaniasis show that the neutrophil effector functions are affected differentially by different species of *Leishmania* parasites.

In conclusion, our results show that patients with active VL in Ethiopia are neutropenic, their circulating neutrophils are highly activated, but have impaired effector functions and an increased frequency of immature neutrophils. We propose that dysfunctional neutrophils play a key role in the severe and systemic inflammatory response characteristic of these patients and therefore contribute to disease severity. More work into the role of inflammation in VL patients and the possible use of anti-inflammatory drugs in combination with anti-leishmanial therapy is warranted.

## Author Contributions

Conceived and designed the experiments: YT, IM, and PK. Performed the experiments: EY, MG, FT, EC, EA, GM, GA, VY, ML, AY, RM, BM, IM, YT, and PK. Analyzed the data: EY, MG, FT, EC, AH, ED, ZS, MMo, MMu, IM, YT, and PK. Wrote the paper: EY, MG, FT, EC, MMo, MMu, YT, IM, and PK.

## Conflict of Interest Statement

The authors declare that the research was conducted in the absence of any commercial or financial relationships that could be construed as a potential conflict of interest.
